# The oxytocin system and early‐life experience‐dependent plastic changes

**DOI:** 10.1111/jne.13049

**Published:** 2021-10-29

**Authors:** Tatsushi Onaka, Yuki Takayanagi

**Affiliations:** ^1^ Division of Brain and Neurophysiology Department of Physiology Jichi Medical University Tochigi Japan

**Keywords:** development, early experience, oxytocin receptor, oxytocin, touch

## Abstract

Early‐life experience influences social and emotional behaviour in adulthood. Affiliative tactile stimuli in early life facilitate the development of social and emotional behaviour, whereas early‐life adverse stimuli have been shown to increase the risk of various diseases in later life. On the other hand, oxytocin has been shown to have organizational actions during early‐life stages. However, the detailed mechanisms of the effects of early‐life experience and oxytocin remain unclear. Here, we review the effects of affiliative tactile stimuli during the neonatal period and neonatal oxytocin treatment on the activity of the oxytocin–oxytocin receptor system and social or emotional behaviour in adulthood. Both affiliative tactile stimuli and early‐life adverse stimuli in the neonatal period acutely activate the oxytocin–oxytocin receptor system in the brain but modulate social behaviour and anxiety‐related behaviour apparently in an opposite direction in adulthood. Accumulating evidence suggests that affiliative tactile stimuli and exogenous application of oxytocin in early‐life stages induce higher activity of the oxytocin–oxytocin receptor system in adulthood, although the effects are dependent on experimental procedures, sex, dosages and brain regions examined. On the other hand, early‐life stressful stimuli appear to induce reduced activity of the oxytocin–oxytocin receptor system, possibly leading to adverse actions in adulthood. It is possible that activation of a specific oxytocin system can induce beneficial actions against early‐life maltreatments and thus could be used for the treatment of developmental psychiatric disorders.

## Graphical abstract

Article Summary: Early‐life experience modulates the activity of the oxytocin system and induces plastic changes in the oxytocin system. Activation of the oxytocin system in early life modulates behaviours in adulthood.

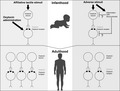

## 1 ROLES OF OXYTOCIN

Oxytocin is mostly synthesized within the hypothalamus and acts mainly on the oxytocin receptor that is expressed in the nervous system and peripheral organs. Oxytocin exerts not only reproduction‐related functions such as delivery of children and milk ejection during the lactation period, but also various actions including control of stress‐related responses, emotional responses and social behaviour.^1^, ^2^, ^3^, ^4^, ^5^, ^6^, ^7^ The oxytocin receptor gene has single‐nucleotide polymorphisms (SNPs) and SNPs of the oxytocin receptor gene have been shown to be associated with difference in pair bonding,^8^ empathy and stress responsiveness,^9^ generous behaviour,^10^ prosocial temperament,^11^ face recognition,^12^ and autism spectrum disorder.^13^

Oxytocin acts within the brain to induce various acute adaptive responses to environmental challenges. Oxytocin exerts neurotrophic actions in some cases. Oxytocin also has organizational effects via acting at certain developmental stages to modulate the developmental trajectory. These oxytocin actions are exerted mainly by activation of the oxytocin receptor located on neurons, astroglia^14^ and microglia^15^ in the nervous system and on cells in peripheral organs.

Various acute actions of oxytocin have been reported. Oxytocin facilitates the acquisition of social recognition^16^ and increases a variety of social behaviours such as parental behaviours (pup retrieval, maternal aggression), mating‐related behaviours, filial huddling, affiliative behaviour, emotional discrimination^17^ and consolation‐related behaviour toward distressed partners.^18^ These actions have been shown to be meditated by enhancement of rewarding effects of social interaction^2^ and by enhancement of attention to social signals.^19^ Oxytocin also acutely attenuates stress‐related or anxiety‐related responses in autonomic, neuroendocrine or behavioural systems by acting on various brain regions, whereas oxytocin appears to facilitate stress responses in socially aversive situations.^20^ In addition, oxytocin has analgesic actions possibly via acting on the spinal cord.^21^, ^22^ Oxytocin has been reported to reduce depression‐related or anxiety‐related behaviour after chronic stress. Oxytocin has negative energy homeostatic actions (decreased satiety, increased energy consumption and hyperthermia)^20^, ^23^, ^24^ partly by inducing meal termination and by activating the sympathetic nervous system. Oxytocin also induces reduction of drug addiction^25^, ^26^ by modulating the activity of the reward system. Oxytocin also has neuroprotective effects via anti‐inflammation actions or switching GABA function from excitation to inhibition.^27^, ^28^, ^29^ Oxytocin also has been shown to play a role in learning and memory including reversal learning.^30^ All of these oxytocin actions of increasing social behaviours, modulating stress‐related responses, inducing negative metabolic states and exerting neuroprotection, have been suggested to contribute to active coping towards various environmental challenges.^20^

In addition to acute actions, oxytocin has been shown to be involved in neurogenesis and neurotropic actions.^31^ Oxytocin administration stimulates neurogenesis in the hippocampus in adult male rats^32^ and conditional ablation of the oxytocin receptor in the hippocampus impairs neurogenesis in mice.^33^

Oxytocin has also been suggested to function during the developmental period.^34^, ^35^ There is accumulating evidence indicating that oxytocin acts in discrete and specific developmental periods to modulate the developmental trajectory of certain neural circuits to control behavioural and neuroendocrine stress responses. These organizational actions of oxytocin during the developmental period may be mediated by acute actions and by neurogenetic actions. On the other hand, both early‐life experience and stimulation of the oxytocin system during early life have been suggested to induce long‐term effects on the oxytocin system and behaviour. However, apparently inconsistent data concerning the effects of early‐life experience and oxytocin on the oxytocin system have been reported. Detailed underlying mechanisms of early‐life experience and oxytocin remain unclear. In the present review, we first discuss the development of the oxytocin–oxytocin receptor system and then effects of tactile stimuli, affiliative parental care, early‐life adverse stimuli and early‐life oxytocin on behaviours and the oxytocin system in adulthood. In the final section, future directions of research concerning organizational actions of oxytocin are discussed.

## 2 THE OXYTOCIN SYSTEM AND EARLY‐LIFE EXPERIENCE

### 2.1 Development of oxytocin neurons and the oxytocin receptor

The oxytocin–oxytocin receptor system appears to be established at a relatively late stage of development compared to the development of the vasopressin system.^36^ Oxytocin mRNA is found at embryonic day 16.5 and the mature form of oxytocin peptide is detected only after birth in mice.^37^ The number of neurons expressing oxytocin immunoreactivity increases during the pre‐weaning period in both mice^35^ and prairie voles.^38^ On the other hand, oxytocin receptor mRNA is detected earlier than oxytocin and is expressed at embryonic day 11–12.5 in mice^37^, ^39^ and rats.^40^ Oxytocin receptor binding is found at embryonic day 16.5 in mice^37^, ^41^ and rats^42^ and increases during the course of development. In some brain regions, expression of the oxytocin receptor is not stable but transient during the postnatal period. These areas include the cingulate cortex,^42^, ^43^ caudate putamen, anterior thalamic nuclei, ventral tegmental area,^40^ septum and auditory cortex.^44^ Sexually dimorphic expression of the oxytocin receptor has been demonstrated in the ventral premammillary nucleus in male mice and in the medial preoptic area in female mice.^45^ It is interesting that oxytocin binding in the ventrolateral part of the ventromedial hypothalamus increases after puberty.^42^, ^45^ The ventromedial hypothalamus has been shown to play roles in aggressive behaviours,^46^, ^47^ sexual behaviour and social defeat posture,^48^ all of which develop after puberty.

Because the oxytocin–oxytocin receptor system develops from the perinatal period until adulthood, experiences especially in early life may influence the development of the oxytocin system to modulate neuroendocrine, autonomic and behavioural responses to environmental challenges in adulthood. In the next section, the effects of tactile stimuli during early‐life stages on behaviours and the oxytocin system are discussed.

### 2.2 Effects of affiliative stimuli including tactile stimulation (touch) in early life

Tactile stimulation plays an important role in non‐verbal emotional communication.^49^, ^50^ Gentle stroking at a slow speed induces a pleasant sensation and social reward via activation of C‐tactile fibres.^51^ Social grooming or allo‐grooming is induced toward distressed conspecifics^52^ to buffer stress responses (social buffering)^53^ and to produce analgesia in the recipients. Social grooming has been suggested to contribute to the formation of social groups. Primates spend a considerable amount of time grooming other individuals to establish and maintain relationships among social members.^54^

During the developmental period, tactile stimulation also plays an indispensable role. Nurturing touch during parental care has been proposed to facilitate the development of emotional and social behaviours and cognitive functions^55^ and to establish an attachment relationship of bonding between infants and their guardians.^49^ Deprivation of touch and social interactions in early childhood leads to irreversible deficits in emotional, social and cognitive behaviours, whereas tactile stimuli with a brush for isolated pups reduce maternal separation‐induced distress in rats^56^ and mandarin voles.^57^ Gentle tactile stimuli during the developmental period establish an affiliative relationship,^58^, ^59^ possibly as a result of the activation of hypothalamic oxytocin neurons.^60^, ^61^, ^62^

Warm sensations in addition to mechanosensation form an important part of tactile stimulation during parental care. Maintenance of body temperature is important for survival during the developmental period when body size is small. Huddling behaviour effectively reduces heat loss. Rodents show thermal‐seeking huddling. It is interesting that oxytocin, which is increased in pups by contact with the mother or parental care,^63^ facilitates the development of thermal‐seeking huddling behaviour.^64^ Oxytocin‐deficient pups select cooler places compared to wild‐type pups in a thermocline performance test.^65^ Furthermore, oxytocin increases thermogenesis via activation of brown adipose tissue.^66^, ^67^ Preference for a warmer place, thermogenesis and possibly increased tolerance of the presence of other individuals by oxytocin may contribute to survival of animals and to the development of social affiliation in early life.

Not only affiliative gentle mechanosensory stimuli^58^, ^59^, ^60^, ^62^ but also affiliative eye contact^68^ acutely activate hypothalamic oxytocin neurons and facilitate oxytocin release.^69^ Parental care including skin‐to‐skin contact with the mother modulates the amount of hypothalamic or serum oxytocin in rat pups.^70^ Rat pups that received frequent licking from their mothers have been reported to show an increased plasma concentration of oxytocin at postnatal day 13,^71^ which is consistent with the view that maternal licking activates the oxytocin system in pups.

As described above, the oxytocin system is acutely activated by affiliative parental care. Parental affiliative stimuli during early life have also been shown to induce long‐lasting influence on the oxytocin system. Early‐life manipulations such as handling, maternal separation, nursery environments and paternal deprivation influence the amount of parental care that pups receive. In the next section, the long‐lasting influence of parental care and its experimental manipulations of handling, maternal separation, cage environment changes and paternal deprivation on the oxytocin system is discussed.

### 2.3 Effects of parental care on the oxytocin system

#### 2.3.1 Effects of parental care or affiliative stimuli on the oxytocin system

Parental care affects expression of the oxytocin receptor in the brain in various animals.^26^, ^72^ Handling during the first week of life in male and female prairie voles facilitates maternal care and increases oxytocin receptor binding in the nucleus accumbens of the offspring^72^ (Table 1), whereas low levels of maternal care are associated with increased oxytocin receptor DNA methylation and thus with decreased expression of the oxytocin receptor.^72^ Positive correlations between parental care received and expression of the oxytocin receptor have also been reported in monogamous prairie voles and rats. Increased parental care has been shown to induce hypomethylation of the oxytocin receptor gene in prairie voles.^73^ Female offspring rats reared by mothers who have a high frequency of pup licking/grooming have been reported to show a high level of oxytocin receptor binding and to exhibit a high level of licking/grooming behaviour toward their own pups,^74^ although no significant difference in the amount of oxytocin receptor mRNA was found in offspring mice of dams that showed high corticosterone and low maternal care.^75^ Increased expression of the oxytocin receptor has been suggested to be mediated, at least in part, via induction of oestrogen sensitivity to express the oxytocin receptor in rats.^76^

**TABLE 1:** Effects of parental care and tactitle stimuli on the oxytocin system in adulthood.

Treatments	Species, sex	Changes in the OXT system	Reference
Early handling (PND 1)‐induced parental care Natural parental care	Male and female prairie voles	OXTR gene methylation ↓ OXTR binding in the Nac ↑ Association of high maternal care and low paternal care with increased OXTR binding in NAc	(72)
Early handling (PND 1)	Male and female prairie voles	OXTR DNA methylation in NAc↓(PND 24)	(73)
Early handling (PND 1, PND 1–7)	Male and female prairie voles	OXT immunoreactivity in the SON but not in the PVN (male) ↑ OXTR binding in the NAc and BNST (female) ↓ OXTR binding in the BNST (male) ↓	(186)
High level of maternal licking/grooming	Male and female rats	OXTR receptor binding in the central AMY, BNST (female but not male) ↑	(74)
Rearing by dams that showed high corticosterone and low maternal care	Male mouse	OXTR mRNA in the frontal cortex, Hip, HYP → Anxiety‐related behaviour, social behaviour, emotional contagion ↓ Aggression ↑	(75)
High level of maternal licking/grooming	Female rats	Oestrogen‐induced OXTR in the MPA, LS ↑	(76)
Post‐weaning tactile stimuli	Male rats	Activity of OXT neurons in the PVN ↑	(58)
Post‐weaning tactile stimuli	Female rats	Activity of OXT neurons in the PVN ↑	(59)

Abbreviations: AMY, amygdala; BNST, bed nucleus of the stria terminalis; Hip, hippocampus; LS, lateral septum; MPA, medial preoptic area; NAc, nucleus accumbens; OXT, oxytocin; OXTR, oxytocin receptor; PND, postnatal day; PVN, hypothalamic paraventricular nucleus; SON, supraoptic nucleus.

Affiliative stimuli received during post‐weaning period also induce long‐lasting stimulating actions on the oxytocin system. Repeated application of gentle stroking stimuli, which induce affiliative behaviour in both female and male rats toward the experimenter, provokes enhanced activity of oxytocin neurons in response to stroking stimuli.^58^, ^59^

#### 2.3.2 Effects of maternal separation on the oxytocin system

Maternal deprivation has been shown in rhesus macaques to increase anxiety in response to isolation, and the sensitivity of maternal separation to induce augmented anxiety depends on a SNP of the oxytocin receptor gene,^77^ suggesting the involvement of the oxytocin receptor in the control of maternal deprivation‐induced emotional behaviour in primates.

Several studies have shown that postnatal maternal separation reduces oxytocin mRNA, oxytocin content or the number of oxytocin neurons in the hypothalamus and decreases the expression of the oxytocin receptor in some brain regions, although differences depending on the species, sex, experimental procedures and brain regions examined have been reported (Table 2).

**TABLE 2:** Effects of maternal separation on the oxytocin system.

Maternal separation	Species, sex	Changes in the OXT system	Reference
15 min day^–1^, PND 2–9	Male rats	Hypothalamic OXT mRNA (PND 9) ↓	(78)
1 min day^–1^, PDN 1–10	Male, female rats	OXT neurons in the parvoPVN ↓ OXT neurons in the magnoPVN and SON →	(79), (80)
15 min day^–1^, PND 1–21	Male rats	OXT content in the AMY (3‐week‐old and adult) and the Hyp (3‐week‐old but not adult) ↓	(81)
6 h day^–1^, PND 1–21	Male rats	OXT content in the AMY (3‐week‐old but not adult) and the Hyp (3‐week‐old but not adult rats) ↓	(81)
3 h day^–1^, PDN 1–14	Male, female mice	OXT immunoreactivity in the PVN (lactating females but not males) ↓	(82)
1 h day^–1^, PND 1–35, 1 h day^–1^, PND 1–35, plus social isolation	Male, female degus	OXT contents in the Hyp (social isolation) ↓ OXT contents in the Hip and PFC ↓	(86)
3 h day^–1^ PND 2‐14	Male rats	OXT mRNA and OXT neurons in the magnoPVN ↓ OXT neurons in the parvoPVN → Restoration by post‐weaning co‐habituation with control	(84)
3 h day^–1^, PDN 1–14	Male mice	OXT neurons in the PVN ↑	(87)
4 h day^–1^, PDN 3–21	Male mice	OXT neurons in the magnoPVN ↓ OXTR (immunoreactivity) in basolateral AMY ↓ Rich environments (PDN22‐120) increase OXT neurons in the parvoPVN and OXTR in the basolateral AMY and prelimbic PFC.	(83)
3 h day^–1^, PND 1–14	Male rats	OXTR binding in the Ins (juvenile [5 weeks], adolescent [8 weeks]), LS (adult [16 weeks]), CP (adult) ↓ OXTR binding in the MPA (adolescent) and VMH (adult) ↑	(88)
4 h day^–1^, PND 1–20	Male rats	OXTR (immunoreactivity) in the medial PFC ↓	(85)
3 h day^–1^, PND 1–21, plus social isolation, forced swimming (15 min) in young adults (PND 62–63)	Male mice	OXTR mRNA in the Hip after 3‐h preweaning stress and social isolation (PND 100 but not in PND 64) ↑	(90)
3 h day^–1^, PND 1–13	Mandarin voles	OXT neurons in the PVN and SON (PND 21) ↓	(187)

Abbreviations: AMY, amygdala; CP, caudate putamen; Hip, hippocampus; Hyp, hypothalamus; Ins, insular cortex; LS, lateral septum; magnoPVN, magnocellular region of the hypothalamic paraventricular nucleus; MPA, medial preoptic area; OXT, oxytocin; OXTR, oxytocin receptor; parvoPVN, parvocelluar region of the hypothalamic paraventricular nucleus; PFC, prefrontal cortex; PND, postnatal day; PVN, hypothalamic paraventricular nucleus; SON, supraoptic nucleus; VMH, ventromedial hypothalamus.

Postnatal maternal separation for a short duration (e.g., 1–15 min day^–1^) has been shown to reduce the hypothalamic oxytocin content in suckling rats^78^ and to reduce the number of hypothalamic oxytocin neurons in adult rats.^79^, ^80^ Reduction of oxytocin content has also been reported in the amygdala of adult male rats.^81^ Maternal separation for a longer duration (e.g., 3 h day^–1^) has also been reported to induce a long‐lasting decrease in oxytocin mRNA or oxytocin content in the hypothalamus and/or in other brain regions in mice,^82^, ^83^ rats^84^, ^85^ and Degus^86^ (but see also Tsuda et al.^87^). Maternal separation has also been shown to reduce oxytocin receptor binding in the lateral septum and the caudate putamen, whereas maternal separation increases oxytocin receptor binding in the ventromedial hypothalamus in rats in adulthood.^88^, ^89^

Interaction between maternal separation and postweaning stresses on expression of the oxytocin receptor has also been suggested. Maternal separation induces no changes in oxytocin receptor mRNA in the hippocampus, while a combination of maternal separation and forced swimming in young adulthood increases oxytocin receptor mRNA in the hippocampus of adult male mice.^90^ The effects of maternal separation on oxytocin receptor expression may be dependent on brain regions and ages when stress is applied.

#### 2.3.3 Effects of limited bedding/nesting materials on the oxytocin system

Living conditions during the pre‐weaning period have been reported to affect the oxytocin system in a sexually dimorphic fashion (Table 3). Male rats under limited bedding and nesting material conditions have been reported to show reduced oxytocin mRNA^91^ and decreased numbers of oxytocin neurons in the hypothalamic paraventricular nucleus and also exhibit reduced oxytocin receptor‐immunoreactive cells in the central amygdala,^92^ although inconsistent data have been reported in female rats (Table 3).

**TABLE 3:** Effects of limited bedding/nesting materials on the oxytocin system.

Duration	Species, sex	Changes in the OXT system	Reference
PND 2–14	Male rats	OXT neurons in the PVN ↓ OXTR cells in the central AMY but not in the basolateral AMY, PVN or dorsal CA3 ↓	(92)
	Female rats	OXT neurons in the PVN↑ OXTR neurons in the central AMY and basolateral AMY but not in the PVN or dorsal CA3 ↑ Reduction of social preference	(92)
PND 7–12	Male rats	OXT mRNA in the magnoPVN and SON (late adolescent) ↓	(91)
	Female rats	OXTR mRNA in the parvoPVN and SON (early adolescent) ↓	(91)

Abbreviations: AMY, amygdala; CA3, hippocampal region CA3; magnoPVN, magnocellular region of the hypothalamic paraventricular nucleus; OXT, oxytocin; OXTR, oxytocin receptor; parvoPVN, parvocelluar region of the hypothalamic paraventricular nucleus; PND, postnatal day; PVN, hypothalamic paraventricular nucleus; SON, supraoptic nucleus.

#### 2.3.4 Effects of paternal deprivation on the oxytocin system

Patterns of parental care, such as biparental care, maternal care only (paternal deprivation) or alloparental care in monogamous animals, have also been shown to affect social behaviours and the oxytocin system in adulthood. In monogamous mandarin voles and prairie voles, both fathers and mothers show parental care toward their children. Paternal absence has been shown to reduce partner preference formation of the offspring in adulthood,^93^ suggesting that the effect of maternal care alone on social behaviour of offspring is different from the effect of biparental care. Paternal deprivation has been shown to reduce oxytocin receptor mRNA and protein or to decrease oxytocin receptor binding in certain brain regions in prairie voles^93^, ^94^ and mandarin voles,^95^, ^96^ although contradictory effects of paternal deprivation on oxytocin neurons have also been reported (Table 4). Consistent with the view that the oxytocin system is involved in effects of paternal deprivation, paternal deprivation‐indued reduction in partner preferences has been shown to be pronounced in prairie voles with a SNP that results in a lower density of the oxytocin receptor in the striatum.^97^

**TABLE 4:** Effects of paternal deprivation on the oxytocin system in monogamous voles.

Species, sex	Changes in the OXT system	Reference
Male and female mandarin voles	OXT neurons in the PVN and SON (PND 14) ↓	(103)
Male and female prairie vole	OXT mRNA in the PVN (female but not male)↑ OXTR binding in the BNST, LS, MPA, central AMY, basolateral AMY →	(93)
Male prairie voles	OXTR binding in the central AMY but not in the BNST, CP, Ins, LS, MPA, NAc, PFC, VMH, or SH (paternal deprivation vs. biparental care) ↓ OXTR binding in the CP, NAc, central AMY (paternal deprivation with alloparental substitution vs. biparental care) ↓	(94)
Female prairie voles	OXTR binding (paternal deprivation vs. biparental care) → OXTR binding in the LS, MPA, NAc (paternal deprivation with alloparental substitution vs. biparental care) ↓	(94)
Male and female mandarin voles	OXTR mRNA in the medial AMY and NAc ↓	(95)
Male and female mandarin voles	PVN OXT neurons projecting to the medial PFC (female)↓ OXTR in the medial PFC (male and female) ↓	(96)
Male and female mandarin voles	OXT neurons in the PVN and SON (PND 21) ↓	(187)
Male and female prairie voles	OXTR binding in the Ins in females (T/T SNP) ↓ OXTR binding in the CP in females (C/T SNP) ↑ OXTR binding in the NAc, CP and Ins in males →	(97)

Abbreviations: AMY, amygdala; BNST, bed nucleus of the stria terminalis; CP, caudate putamen; Ins, insular cortex; LS, lateral septum; MPA, medial preoptic area; NAc, nucleus accumbens; OXT, oxytocin; OXTR, oxytocin receptor; PFC, prefrontal cortex; PND, postnatal day; PVN, hypothalamic paraventricular nucleus; SH, septohippocampal nucleus; SON, supraoptic nucleus; T/T (C/T) SNP, T/T (C/T) variant of oxytocin receptor gene intron single nucleotide polymorphism NT213739; VMH, ventromedial hypothalamus.

As summarized above, parental care has been shown to produce long‐lasting changes in the oxytocin system. Generally, oxytocin neuronal activity is activated after affiliative parental care and oxytocin receptor expression is increased in correlation with the amount of paternal care, although differential influences dependent on brain regions, experimental manipulation and sex have been reported.

Oxytocin neurons have been shown to be activated not only by affiliative stimuli, but also by stressful stimuli in adulthood. During the developmental period, stressful stimuli as well as parental care acutely influence activity of oxytocin neurons. Stressful stimuli in early life have also been shown to induce long‐lasting changes in the oxytocin system,^26^, ^98^ as is the case for the long‐lasting changes induced by parental care as described above. In the next section, the effects of adverse manipulations in early life (prenatal, postnatal and post‐weaning periods) on the oxytocin system are discussed.

### 2.4 Effects of sensory deprivation and stressful stimuli in early life on the oxytocin system

In animals in adulthood, various stressful stimuli,^99^ regardless of whether given in a social context such as a social defeat paradigm^48^ or in a non‐social context such as a conditional fear paradigm,^20^, ^100^ have been shown to acutely activate hypothalamic oxytocin neurons and facilitate oxytocin release. It has also been shown in humans that psychological stress increases plasma oxytocin concentrations.^101^ By contrast to the acute effects of stressful stimuli, chronic stressful stimuli might produce different actions. In female mandarin voles, chronic social defeat stress has been reported to induce anxiety‐related and depression‐related behaviour associated with a reduction of oxytocin fibres and oxytocin receptor mRNA in the nucleus accumbens. Administration of oxytocin into the nucleus accumbens has been shown to reverse the anxiogenic and depressive actions of chronic defeat stress.^102^

During the developmental period, stressful stimuli influence oxytocin neurons in the hypothalamus. Social isolation stress during the lactating period has been reported to modulate oxytocin immunoreactivity in the hypothalamus of monogamous mandarin voles.^103^

The oxytocin system has been shown to be influenced for a long time after early‐life stressful stimuli including prenatal exposure to alcohol (Table 5). Prenatal stress has been shown to decrease the number of oxytocin neurons in the magnocellular paraventricular nucleus in the adult offspring of dam rats.^104^

**TABLE 5:** Effects of pre‐weaning stress and post‐weaning social isolation on the oxytocin system.

Stressful stimuli	Species, sex	Changes in the OXT system	Reference
Prenatal alcohol (ethanol liquid diet GD 5–20), nicotine (3–6 mg kg^–1^ day^–1^, GD 4‐PND 1)	Male and female rats	OXT in the VTA (adolescent male) ↑, (Adult male) ↓ OXT in the MPA, AMY, Hip →	(188)
Prenatal alcohol (ethanol liquid diet GD 5–20), nicotine (3–6 mg kg^–1^ day^–1^, GD 4 to PND 10)	Male and female rats	OXT mRNA in the PVN and SON → OXTR binding in the NAc and CA3(male) ↑ OXTR binding in the VMH →	(189)
Prenatal alcohol (4.5 g kg^–1^ ig GD 1–22 (dams), 3 g kg^–1^ ig PND 2–10)	Male and female rats	OXTR binding in the AMY (male) → (↓ vs. non‐treatment control) OXTR binding in the AMY(female) ↓	(190)
Liquid ethanol diet, GD 1–21	Male rats	OXTR binding in the mPFC and central AMY ↑ (early adolescent), LS and NAc (late adolescent) ↑ OXT mRNA in the magnoPVN, parvoPVN and SON (late adolescent, male) ↓ OXT mRNA in the SON (late adolescent, female) ↓	(191) (91)
Prenatal stress (30 min day^−1^ restraint of dams during the last 7 days)	Male and female rats	OXT neurons in the magnoPVN but not in the parvoPVN or SON (male but not female) ↓	(104)
Postnatal sensory deprivation from birth (whisker deprivation, dark rearing)	Male and female mice	OXT mRNA in the Hyp, OXT in the sensory cortex ↓ Rich environments increased OXT mRNA in the Hyp and OXT in the cortex	(105)
Post‐weaning social isolation (PND 24 until 12 or 13 weeks)	Male and female rats	OXT neuron activity in the PVN and SON to novel conspecifics (female not male) ↓ OXT neurons in the PVN and SON (male and female) →	(106)
Post‐weaning social isolation from postnatal 6 weeks for 5 weeks	Male mice	OXT mRNA in the PVN → OXTR mRNA in the central AMY ↓	(107)
Post‐weaning social isolation (PND 21–74)	Male and female rats	OXT mRNA in the PVN ↑ OXT mRNA in the SON → OXTR binding in the anterior NAc ↓ OXTR binding in the BNST (females) ↓ OXTR binding in the LS, central AMY, VMH →	(108)
Chronic social defeat for 14 days (PND 70)	Female mandarin voles	OXT fibres, OXTR immunoreactivity, OXTR mRNA in the NAc but not in CA1 and CA3 ↓	(102)

Abbreviations: AMY, amygdala; BNST, bed nucleus of the stria terminalis; CA1, hippocampal region CA1; CA3, hippocampal region CA3; GD, gestational day; Hip, hippocampus; Hyp, hypothalamus; ig, intragastrical administration; LS, lateral septum; magnoPVN, magnocellular region of the hypothalamic paraventricular nucleus; MPA, medial preoptic area; mPFC, medial prefrontal cortex; NAc, nucleus accumbens; OXT, oxytocin; OXTR, oxytocin receptor; parvoPVN, parvocelluar region of the hypothalamic paraventricular nucleus; PND, postnatal day; PVN, paraventricular nucleus; SON, supraoptic nucleus; VMH, ventrolateral hypothalamus; VTA, ventral tegmental area.

Postnatal sensory deprivation has also been shown to decrease activity of oxytocin neurons in the hypothalamus. Sensory deprivation by whisker deprivation and dark rearing have been reported to decrease oxytocin mRNA in the hypothalamus and to reduce excitatory synaptic transmission in the sensory cortex in mice.^105^ Oxytocin administration has been shown to rescue this impairment, suggesting an important role of oxytocin in early experience‐dependent cortical development.^105^

Post‐weaning social isolation has also been shown to reduce activity of oxytocin neurons in the hypothalamus of female rats in response to exposure to novel conspecifics, to impair social preference^106^ and to reduce oxytocin receptor mRNA in the amygdala in mice.^107^ Oxytocin receptor binding has also been shown to be reduced in the nucleus accumbens in rats, although hypothalamic oxytocin mRNA has also been reported be increased in rats^108^ (Table 5).

As discussed above, numerous animal experiments have shown that various stressful treatments during early‐life stages have long‐lasting inhibitory actions on the oxytocin system. In the next section, the effects of adverse environments in early life on the oxytocin system in humans are discussed.

### 2.5 Effects of adverse environments in early‐life stages on the oxytocin system in humans

In humans, prenatal or postnatal stressful stimuli have been shown to be associated with altered functions of brain regions including the superior frontal gyrus for cognitive processing, amygdala for emotional processing, precuneus for memory processing and putamen for reward‐related learning processing.^109^ Early‐life adversities have been reported to be associated with vulnerability to a variety of diseases in adulthood including depression,^110^ post‐traumatic stress disorder, anxiety disorders, substance use disorders, cardiovascular disease,^111^, ^112^, ^113^, ^114^ chronic pain^115^ and irritable bowel syndrome.^116^ Associations of SNPs of the oxytocin receptor gene with psychiatric disorders dependent on early‐life experience (parental care and abuse)^117^, ^118^, ^119^, ^120^, ^121^, ^122^, ^123^, ^124^ and with susceptibility to structural brain changes after childhood emotional neglect^125^ have been demonstrated. Women with an oxytocin receptor gene SNP of high expression of the oxytocin receptor have been reported to be more susceptible to childhood maltreatment‐related impairments in maternal behaviours.^126^

Childhood maltreatment has been shown to increase DNA methylation and to modulate the expression of various genes^127^ including the oxytocin receptor gene. DNA methylation of the oxytocin receptor gene has been shown to be linked to social behaviour, emotional behaviour and psychiatric diseases.^128^, ^129^ A meta‐analytical study in humans has shown that adverse environments during early life tend to induce lower activity of oxytocin neurons and lower expression of the oxytocin receptor as a result of a higher level of oxytocin receptor gene methylation.^130^ Children or adolescents who have experienced maltreatment have been reported to show greater methylation of the oxytocin receptor gene in saliva samples, and a negative association between volume of the left orbitofrontal cortex and methylation was found.^131^ In adult women, an inverse association between severity of childhood abuse received and oxytocin concentrations in cerebrospinal fluid has been shown,^132^ although a positive correlation between urine oxytocin and less severe maltreatment during childhood was also reported.^133^ On the other hand, several studies have found no direct association between childhood abuse and methylation of the oxytocin receptor gene.^134^, ^135^ Maltreatment has also been shown to be associated with oxytocin receptor gene methylation indirectly via social instability.^134^ Childhood abuse has been reported to induce anxiety and depression in a manner dependent on DNA methylation of the oxytocin receptor gene.^135^ It has also been shown that oxytocin receptor gene methylation was transmitted from mothers to their newborns and that the intergenerational transmission of methylation levels was not observed in mothers who experienced maltreatment during childhood.^136^ The findings suggest that experience of maltreatment disrupts possibly adaptive intergenerational epigenetic transmission.

Early‐life experience has also been shown to modulate effects of oxytocin administration possibly by inducing changes in the oxytocin system in adulthood. The effects of oxytocin administration appear to be different depending on early‐life stress in humans. Intranasal oxytocin application reduced premenstrual emotional symptoms in a control group of females, whereas oxytocin administration augmented the emotional symptoms in women who had experienced early‐life abuse.^137^ Oxytocin has been found to induce anxiolytic actions during stressful conditions in the presence of a friend only in females who have experienced a high level of childhood adversity.^138^ Considering the notion that oxytocin has anxiolytic actions in neutral ambiguous conditions, whereas it induces anxiogenic actions in subjectively adverse conditions by increasing the salience of social stimuli, early‐life maltreatments shift neutral levels of emotional states to change actions of oxytocin in adulthood.

As discussed in the above two sections, both animal and human studies have shown that stressful stimuli in early life acutely activate the oxytocin system and induce long‐lasting suppressive actions on the oxytocin system. In the next section, the role of oxytocin and effects of oxytocin administration in early life is discussed.

### 2.6 Long‐term effects of early‐life oxytocin stimulation

Oxytocin in early life has been shown to attenuate early‐life stress‐induced or genetically induced impairments of social behaviour or energy metabolisms, although detailed mechanisms of the organizational actions of oxytocin remain to be clarified.

#### 2.6.1 Prenatal oxytocin stimulation

The prenatal oxytocin system has been suggested to affect behaviour in adulthood. Male oxytocin‐deficient mice born to oxytocin‐knockout dams and fostered to wild‐type dams show augmented aggressive behaviour, whereas oxytocin knockout male mice born to heterozygous dams and fostered to wild‐type dams do not show heightened aggressive behaviour.^39^, ^139^ On the other hand, male oxytocin receptor‐deficient mice that have never been stimulated by oxytocin during their life show augmented aggressive behaviour. These findings suggest that oxytocin during the prenatal or perinatal period reduces aggressive behaviour in adulthood. Consistent with this view, prenatal administration of an oxytocin antagonist has been shown to facilitate later aggressive behaviour in male mice.^140^

#### 2.6.2 Perinatal oxytocin stimulation

Perinatal oxytocin has also been suggested to affect behaviour in adulthood. At the time of delivery, a large amount of oxytocin is released in mothers and might act in both mothers and newborns.^141^ A part of the oxytocin released in mothers may be delivered into newborns because the placenta can partly transport oxytocin.^142^ Perinatal oxytocin has been proposed to protect the newborn brain by inducing a hyperpolarizing response to GABA.^27^, ^28^, ^29^

Plasma oxytocin concentrations are lower in caesarean‐section neonates and mothers than in vaginally born babies and mothers.^143^ The effects of neonatal oxytocin administration to caesarean‐section delivered pups have been reported in animals. In mice, caesarean section has been shown to reduce preference to maternal bedding during the preweaning period and to impair social recognition in adulthood. Postnatal daily administration of oxytocin (0.2 or 2 µg, s.c. administration, postnatal days 1–5) has been reported to increase plasma oxytocin in mice in adulthood, and a high dose of postnatal oxytocin administration rescued caesarean‐section induced impairment in preweaning maternal bedding preference and a lower dose of postnatal oxytocin administration resulted in recovery of impaired social recognition in adulthood.^144^ Caesarean‐section delivery has also been shown to be associated with a heightened risk for developing obesity.^145^, ^146^ Postnatal oxytocin administration (1 mg kg^–1^, postnatals day 10–14) has been reported to increase^147^ or decrease^148^ body weight in rats. Further studies are necessary to clarify the role of early‐life oxytocin in metabolism in adulthood.^149^

Oxytocin has been widely used for stimulation of births. Prenatal oxytocin administration to mothers has been shown to affect various brain functions in adulthood. In prairie voles, pups who received exogenous oxytocin via administration to their mothers were reported to show increased plasma oxytocin and increased activation of oxytocin neurons, suggesting that maternally administered oxytocin crosses the placenta into the pup and activates hypothalamic oxytocin neurons of the pup. Adult male offspring of oxytocin‐administered dams have been reported to show increased expression of the oxytocin receptor in the central amygdala and insular cortex, to exhibit increased alloparental caregiving toward pups and to display close social contact with other adults.^150^ The effects of neonatally administered oxytocin on alloparental behaviour and partner preference behaviour in adulthood have also been reported be different dependent on oxytocin dosages in female prairie voles.^151^ Neonatal oxytocin at some dosages has been reported to facilitate alloparental behaviour and partner preference in adulthood, whereas neonatal oxytocin at a higher dosage has been shown to impair these social behaviours.

For stimulation of labour in humans, low doses of oxytocin are applied over a long period. In humans, a population‐based study showed that perinatal exogenous administration of oxytocin has no association with childhood emotional disorders,^152^ and the relationship between oxytocin administration and postpartum depression also remains unclear.^153^ However, a case–control study showed an association between a higher dose or longer duration of oxytocin administration and the odds of developing autism spectrum disorder,^154^ and a positive relationship between oxytocin administration and a higher risk of postpartum depression has been reported.^155^ Further investigations are necessary.

#### 2.6.3 Postnatal oxytocin stimulation

Milk contains oxytocin.^156^ Orally administered oxytocin has been shown to be absorbed into the peripheral circulation, especially in neonates.^157^ The oxytocin receptor exits in peripheral organs including the intestinal system.^158^ A small amount of oxytocin may penetrate the blood–brain barrier into the brain. The effects of oxytocin in milk remain to be clarified.

On the other hand, postnatal oxytocin application at large dosages has been reported to rescue social behaviour, pain‐related behaviour and memory in adulthood, which are impaired by genetic manipulations or behavioural treatments.

Postnatal daily administration of oxytocin (2 µg day^–1^ in the first 2 days or postnatal week) has been shown to increase pre‐ or post‐synaptic transcript levels of neurexins and neuroligins^159^ and to prevent deficits in social behaviour and learning abilities in adult Magel2‐deficient mice, a mice model of Prader–Willi syndrome and autism spectrum disorder.^160^ Intranasal oxytocin administration (200 µg kg^–1^) during postnatal days 7–21 has also been shown to restore the number of hypothalamic oxytocin neurons and to reverse social deficits in contactin‐associated protein‐like 2 (CNTNAP2)‐deficient mice, another model of autism spectrum disorder.^161^ Postnatal oxytocin administration (3 µg day^–1^ in the first week) has also been reported to result in recovery of a decrease in oxytocin neurons and deficits in social behaviour in rats that had been prenatally treated with valpronic acid.^162^

Neonatal maternal separation (3 h per day during postnatal days 2–12) in rats has been shown to induce hypersensitivity in response to nociceptive stimuli and to impair oxytocin receptor‐dependent stress‐induced analgesia and oxytocin receptor‐dependent anti‐hyperalgesia.^163^ Because maternal separation does not change oxytocin receptor mRNA in the spinal cord, it is likely that maternal separation acts at supraspinal levels such as on hypothalamic oxytocin neurons to impair oxytocin‐induced analgesia. Interestingly, the effect of neonatal maternal separation on increased nociceptive response is attenuated by neonatal oxytocin application.^163^

Neonatal isolation (3 h day^–1^ during postnatal days 1–14) reduces partner preference in adult female prairie voles, and female voles that show high oxytocin receptor binding in the nucleus accumbens have resilience to neonatal isolation‐induced impairments. These social isolation‐induced impairments in attachment behaviour are mitigated by neonatal treatment with melanotan‐II administration, which facilitates oxytocin release.^164^ Oxytocin administration in male prairie voles has also been shown to increase partner preference and to decrease anxiety‐related behaviour, whereas the administration of an oxytocin receptor antagonist decreases parental behaviour in adulthood.^165^

Chronic stress in early life caused by maternal separation (3 h day^–1^ from postnatal days 1–21) induces reduction in spatial memory and disturbance of long‐term potentiation in the hippocampus, and this reduction has been shown to be attenuated by repetitive application of oxytocin in rats.^166^

#### 2.6.4 Long‐term effects of oxytocin stimulation on the brain

Early‐life experience influences the oxytocin system and oxytocin simulation in early life induces long‐lasting effects on not only the oxytocin system, but also other brain circuits to affect emotional or social behaviours in adulthood. However, the underlying mechanisms of oxytocin actions remain largely unknown.

Oxytocin receptor activation upregulates the activity of potassium‐chloride co‐transporter2 (KCC2) to switch GABA function from depolarization to hyperpolarization in an early and narrow developmental time window of life.^29^ Neonatal oxytocin has also been shown to modulate vasopressin V1a receptor binding in the bed nucleus of the stria terminalis, cingulate cortex, mediodorsal thalamus, medial preoptic area and lateral septum in prairie voles in adulthood.^167^ Early‐life oxytocin administration also influences alpha2 receptor agonist binding in rats of adulthood.^168^ Expression of the oestrogen alpha receptor has also been reported to be changed by neonatal oxytocin administration in a manner dependent on sex, brain regions and oxytocin dosages in prairie voles.^169^ Neonatal oxytocin administration also influences serotoninergic innervation in the anterior hypothalamus, cortical amygdala and ventromedial hypothalamus in male prairie voles.^170^ Expression of brain‐derived neurotrophic factor in the hippocampus of rats has also been shown to be modulated by oxytocin dependent on sex and age.^171^ The effects of oxytocin on glutamatergic system in the hippocampus.^159^ and in the prefrontal cortex^172^ have also been shown. Neurogenetic actions by oxytocin may also be involved in the long‐lasting actions of early‐life oxytocin.

## 3 FUTURE DIRECTIONS OF RESEARCH AND CONCLUSIONS

**FIGURE 1:**
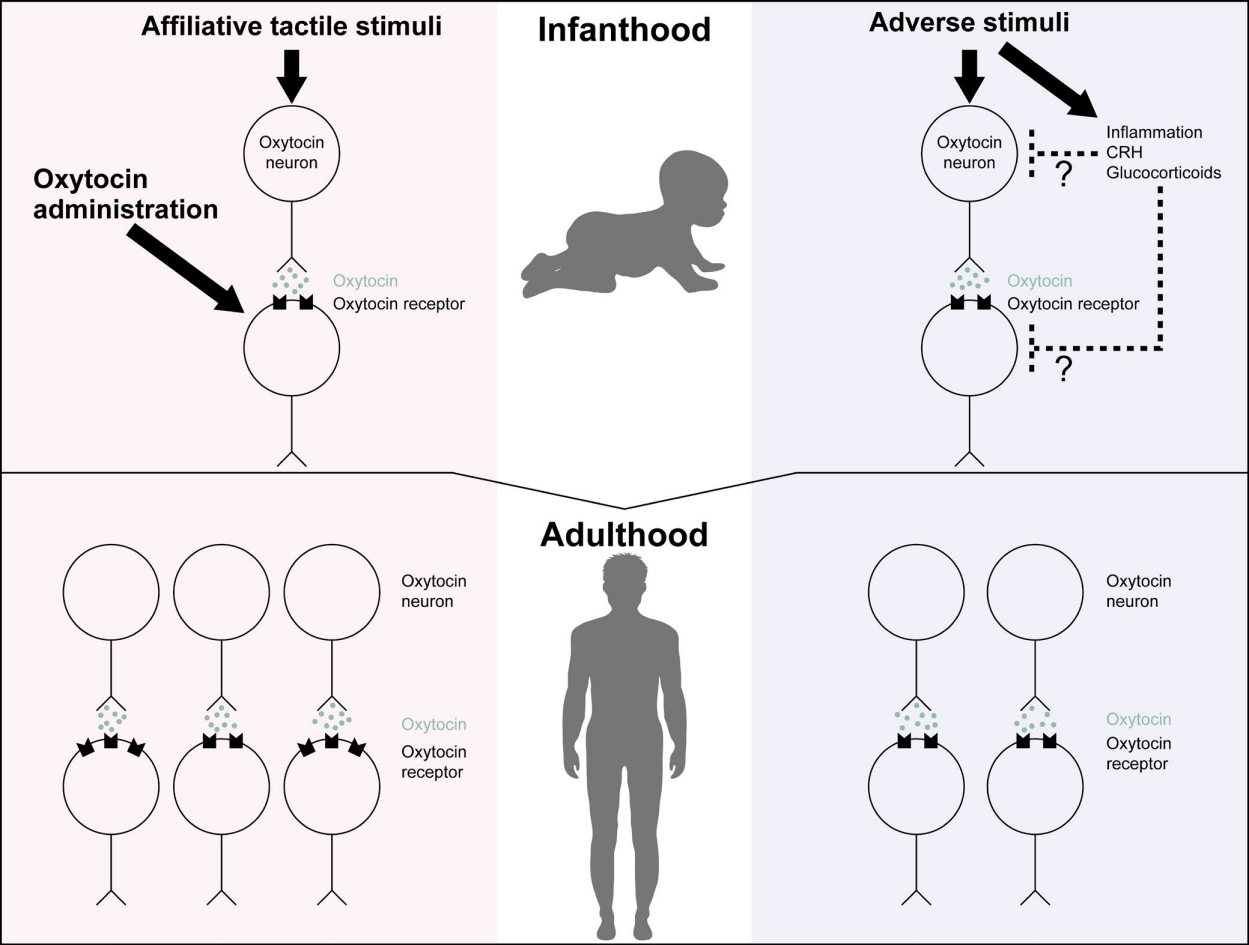
Effects of early‐life stress on the oxytocin–oxytocin receptor system. Affiliative tactile stimuli and oxytocin administration activate the oxytocin system in infanthood and induce high levels of activity in some brain regions to facilitate social behaviour and induce anti‐anxiety actions in adulthood. Early‐life adverse stimuli activate the oxytocin system acutely in infanthood but tend to induce lower levels of activity in some brain regions in adulthood. CRH, corticotropin‐releasing hormone

Early‐life experience changes the activity of oxytocin neurons and possibly oxytocin receptor‐expressing neurons at the time of experience, resulting in plastic changes in the expression of oxytocin and/or the oxytocin receptor in adulthood, although the magnitude and direction of changes appear to be dependent on early experience, genetic differences, brain regions and sex. Generally, affiliative experience activates the oxytocin system in early life and induces enhanced activity of oxytocin neurons and increased expression of the oxytocin receptor in adulthood. On the other hand, early adverse experience activates the oxytocin system in early life but induces reduced activity of oxytocin neurons and the oxytocin receptor in adulthood. The mechanisms underlying the opposite long‐lasting effects of affiliative and adverse experiences remain to be clarified. Oxytocin administration at moderate dosages in early life induces activation of the oxytocin system in adulthood, suggesting that the oxytocin system in early life has a positive feedback system to induce long‐lasting activation of the oxytocin system in adulthood. On the other hand, administration of a large amount of oxytocin in early life sometimes induces opposite actions in adulthood. The magnitude of activations of oxytocin neurons by affiliative stimuli is relatively small and the activation is transient, whereas stressful stimuli can drastically activate oxytocin neurons. Stressful stimuli stimulate not only the oxytocin system, but also other systems including the hypothalamic‐pituitary‐adrenocortical axis, sympatho‐adrenomedullary system, opioid system, allopregnanolone system and inflammatory responses. It is possible that overactivation of oxytocin neurons and/or other stress‐related systems activated during stressful stimuli in early‐life stages induces long‐lasting inhibitory actions on the oxytocin system in adulthood (Figure 1).

The effects of early‐life stimuli and oxytocin administration have been shown to be dependent on sex and brain regions examined. Female pups appear to be more sensitive than male pups to the neonatal manipulation of oxytocin,^173^ although sex disparities should be interpreted cautiously.^174^ The finding that early‐life experience modulates the brain differentially depending on sex and brain region are not surprising. Expression of the oxytocin receptor in some brain areas is sexually dimorphic,^45^ and oestrogens or testosterone modulate the expression of oxytocin and the oxytocin receptor.^2^ The oxytocin–oxytocin receptor system has differential roles depending on the brain region with respect to the control of behaviour and autonomic systems.^2^, ^20^ It is important to clarify region‐specific roles of the oxytocin system using modern genetic tools that can manipulate activity of the oxytocin system in region‐ and/or function‐specific manners.^4^ Oxytocin is released from not only axon terminals, but also non‐synaptic axonal varicosities,^175^ cell bodies and dendrites^176^ with different temporal patterns. Visualization of locally released oxytocin in specific brain regions with high time resolution using new oxytocin sensors^177^, ^178^, ^179^ is also useful for clarification of local oxytocin functions.

Inconsistent and sometimes contradictory data concerning the effects of early‐life experience and oxytocin administration have been reported. In an epigenetic landscape model, balls, which represent the brain, stand at a similar initiating point and roll downhill through bifurcating valleys, resulting in the balls reaching very different endpoints. Thus, small differences in experimental procedures for early‐life experience among different laboratories might have caused a large variation in the data for adulthood.

It is also likely that discrepancies are caused, at least in part, by the complex nature of early‐life experience. For example, maternal separation has several components including sensory deprivation from mothers (loss of touch, warmth, olfactory signals, visual signals of the mother and littermates) and also exposure to novel environments during separation, possible nutritional deprivation and temperature loss. Maternal separation modulates the parental care of mothers afterwards. Some of these components may be variable depending on laboratories. Simpler stimulation of a mono‐sensory system and pathway‐specific activity manipulation using molecular techniques may be useful as an early‐life stimulus for clarifying the effects of early‐life experience.

Oxytocin administered not only during the neonatal period, but also during the adolescent period has been shown to have long‐lasting prosocial, anti‐anxiety and anti‐aggression actions.^180^, ^181^, ^182^ During the adolescent period, the release of sexual hormones is drastically increased and social behaviours among peers are developed. The roles of the endogenous oxytocin system, the activity of which is modulated by sexual hormones and which also facilitates social behaviour, during the adolescent period remain to be clarified.^183^

Oxytocin has been considered for treatment of autism spectrum disorder^184^ and post‐traumatic stress disorder.^185^ From translational points of view, it is important to clarify the long‐term actions of oxytocin during early life and to find ways for activation of functionally specific oxytocin pathways to induce beneficial actions of the oxytocin system. It is possible that selective activation of a specific oxytocin system would rescue the adverse actions of early‐life stressful experience and thus could be used for the treatment of developmental psychiatric disorders.

## CONFLICT OF INTERESTS

The authors declare that they have no conflicts of interest.

## AUTHOR CONTRIBUTIONS

**Tatsushi Onaka:** Conceptualization; Funding acquisition; Writing – original draft; Writing – review & editing. **Yuki Takayanagi:** Conceptualization; Funding acquisition; Writing – review & editing.
